# Caregivers’ knowledge and attitudes about childhood diarrhea among refugee and host communities in Gambella Region, Ethiopia

**DOI:** 10.1186/s41043-018-0156-y

**Published:** 2018-11-22

**Authors:** Getachew Kabew Mekonnen, Bezatu Mengistie, Geremew Sahilu, Worku Mulat, Helmut Kloos

**Affiliations:** 1 Ethiopian Institute of Water Resources, Addis Ababa University, P.O. BOX. 150461, Addis Ababa, Ethiopia; 2 College of Health and Medical Sciences, Haramaya University, P.O. Box 1570, Harar, Ethiopia; 3 Department of Civil and Environmental Engineering, University of Connecticut, Storrs, CT USA; 4 Department of Epidemiology and Biostatistics, University of California, San Francisco, CA USA

**Keywords:** Acute diarrhea, Knowledge, Attitudes, Under-five children, Refugees

## Abstract

**Background:**

Maternal knowledge, attitudes, and practices related to hygiene, breastfeeding, sanitary food preparation, and appropriate weaning practices are potentially important determinants in the occurrence of diarrhea in children. However, few studies have been carried out about the knowledge and attitudes about childhood diarrhea among parents in refugee camps and host communities.

**Objective:**

This study aims at assessing the caregivers’ knowledge and attitudes regarding acute diarrhea in under-five children among refugee and host communities in Gambella Region, Ethiopia.

**Methodology:**

This cross-sectional study, employing multistage sampling, was carried out from September to December 2016. Data was collected by a questionnaire-based interview, and 1667 caregivers were included in this study. A composite knowledge score was calculated, and a five-point Likert type of attitude scale was developed to assess the attitudes of the caregivers towards childhood diarrhea. Appropriate descriptive statistics and logistic regression models were used. Odds ratios (ORs) are presented with their 95% confidence intervals (CIs), and all analyses were performed at the 5% significance level (*p* < 0.05).

**Result:**

The study indicates that 633 (28.0%) of the caregivers had poor knowledge, while 393 (23.6%) of them had unfavorable attitudes towards childhood diarrhea. Knowledge of the caregivers was significantly associated with formal education (AOR, 1.3; 95% CI, 1.03–1.5) and health information obtained from a health care institution (AOR, 1.8; 95% CI, 1.28–2.3). Caregivers’ knowledge is a single predictor of their attitude (*p* < 0.001), and Pearson’s correlation coefficient revealed that there was a significant positive correlation (*r* = 0.2, *p < 0.001*) between knowledge and attitude scores.

**Conclusion:**

The study indicates that significant numbers of caregivers had inadequate knowledge and unfavorable attitudes about diarrhea in under-five children. Designing and implementing an inclusive health education intervention focusing on uneducated child caregivers may be beneficial for improving knowledge and attitudes towards reducing the incidence of acute childhood diarrhea in the region.

## Introduction

Acute diarrhea is one of the most common causes of childhood morbidity and mortality in developing countries [1, 2]. Diarrhea accounts for 760,000 deaths in children under 5 years of age worldwide, representing 15.2% of all deaths among children less than 5 years of age in developing countries [3]. A high proportion of child morbidities and 25% of all deaths in refugee populations are due to diarrhea [4]. The majority (60–70%) of diarrhea-related deaths are caused by dehydration due to loss of water and electrolytes [5]. The Integrated Management of Childhood Illness guidelines recommend the use of ORT along with continued feeding for appropriate diarrhea case management [6]. Recently, ORS fluid replacement accompanied by zinc treatment became the most successful approach [7], and appropriate antibiotics are also required to effectively treat bacterial diarrhea [8–10]. For these reasons, maternal knowledge and perceptions related to hygiene, breastfeeding, sanitary food preparation, and appropriate management and weaning practices are important determinants in the occurrence of diarrhea in children [11, 12]. Mothers’ basic knowledge about diarrhea depends on their educational status, their prior experience of managing the disease, and their culture, among others [13]. Mothers in marginalized communities have been found to have poor knowledge and attitudes about diarrhea in children [14]. Most mothers in one rural community did not recognize signs of dehydration due to diarrhea [15, 16], and many of them are unaware of fluid replacement or ORS use in treating diarrhea [17].

Since mothers are the chief caregivers of children, their socioeconomic condition significantly influences the health status of their children and outcome of diarrhea episodes [18]. Lack of caregivers’ knowledge and awareness usually results in poor use of available information on preventing and managing childhood diarrhea in developing countries [19]. Caregivers’ knowledge and attitudes are associated with socio-demographic conditions, culture, access to health education, and others [13, 20, 21]. Despite the universal popularity of oral rehydration solution in preventing child dehydration due to diarrhea, ORS is underutilized and incorrectly used, which usually resulted from the lack of mothers’ knowledge [19] or perceptions of the seriousness of diarrhea [22]. Some studies showed that mothers have the intention to reduce and even stop fluids during diarrhea [23]. These attitudes and practices may be aligned to caregivers’ knowledge and perceptions towards preventing childhood diarrhea [24]. Largely due to wide range of predisposing factors, diarrheal disease burden is not uniform in different regions of the world.

Diarrhea remains a major problem in refugee camps and rural communities in sub-Saharan Africa [25, 26]. The problem may be aggravated by political instability in countries where refugees originated, including South Sudan, Somalia, and Eritrea [27]. Hence, identifying knowledge gaps is critical for the development of effective preventive programs. To our knowledge, no formative studies previously have been undertaken on caregivers’ knowledge and attitude about childhood diarrhea in refugee camps in Ethiopia. Thus, this study was aimed to assess caregivers’ knowledge and attitude of caregivers regarding diarrhea in under-five children to generate pragmatic information in order to guide and influence public health policies in the region.

## Methodology

### Study area and design

The cross-sectional study was carried out from September to December 2016 in Pugnedo and Terkiedi refugee camps and the host in Gambella Region. Gambella is one of the 11 administrative regions of Ethiopia located along the Sudan border west of Addis Ababa. Multistage sampling was employed to select the study households. The objective of this study was to assess the caregivers’ knowledge and attitudes regarding childhood diarrhea among refugee and host communities in Gambella Region, Ethiopia.

### Sample size determination

The sample size was determined considering a 43% prevalence of diarrhea among children under five in internally displaced South Sudanese [28] and 31% 2-week period prevalence of childhood diarrhea morbidity in rural communities in southwestern Ethiopia [29], representing the host communities, with 80% power, 95% confidence level, 1.5 design effect, and 10% non-response, and the final sample doubled the efficacy of a stratified community data analysis. The total sample size was determined to be 1782 (891 each from the refugee and host communities). The number of households with under-5-year-old children was 10,085 in Pugnido and 9,863 in Teirkidi refugee camps. Gog District, located near the refugee camps, was selected based on the potential to minimize confounding geographical factors. An equal number of study subjects were allocated to the two types of the communities, and samples were distributed proportionally to the size of the target population. Each household with under-5-year-old children was selected by systematic random sampling techniques (every 21st and 4th in the refugee and host communities, respectively).

### Data collection method

This study was carried out among caregivers who had at least one under-five child; data were collected using a questionnaire during face-to-face interviews. The questions pertain to socio-demographic characteristics of households, caregivers’ knowledge and attitudes about diarrhea, the predominant household drinking water source, availability of a latrine, and diarrheic condition of the child. It consisted of 13 open and closed questions and was divided into section A, which had five multiple choice knowledge questions, and section B, which had eight questions on attitudes.

The knowledge tool contained questions about the definition (1 point), causes (6 points), impacts (4 points), the management (8 points), and prevention (6 points) aspects of diarrhea. One point was given for each correct answer and a score of zero for wrong or uncertain answers. Each of the knowledge questions had one or more correct answers, and all questions had a total of 25 correct answers (or points). The caregivers’ knowledge about diarrhea is indicated by the total points. A composite knowledge score was calculated, with higher scores indicating more correct answers. Mothers scoring above average were considered to have adequate knowledge, and mothers with a score below average were considered as having poor knowledge. The scores below 13, 13 to 19, and more than 19 were classified as low, average, and good knowledge, respectively.

The attitude questions covered caregivers’ perceptions of a child contracting diarrhea regularly, preventing diarrhea by hand washing using water and soap, washing hands after toilet use, washing hands before eating, drinking clean water, exclusive breastfeeding, vaccination, and treating diarrhea with ORS. A five-point Likert type of attitude scale was developed to assess the attitudes of the caregivers towards childhood diarrhea. The scoring for each correct answer was given as: 1 = strongly disagree, 2 = disagree, 3 = neutral, 4 = agree, and 5 = strongly agree. Negative scores were given for the incorrect answers. These eight attitude questions carried 8 to 40 points for each interviewee. Attitude scores less than 25 were classified as unfavorable, and scores 25 (50%) and above were considered as favorable.

### Data quality control measures

The questionnaire was developed in English language and then translated into the local Nuer and Agnwak languages for better communication with the study subjects. The recruited data collectors were those who completed at least their secondary education and are able to write, read, and understand English well. Training was given by the principal investigator a week before the onset of the study. Additionally, the questionnaire was pre-tested on 50 households of a similar community in the Jawi refugee camp, and necessary corrections were made accordingly. We checked the data onto consistency and completeness. The reliability test with Cronbach’s alpha coefficient for the knowledge and attitude questions was 0.75 and 0.84, respectively.

### Data analysis

The responses were coded, entered, cleaned, and analyzed using STATA Version 14. Appropriate descriptive statistics such as mean, range, standard deviation, frequency, and percentages were calculated. Two sample *t* test was used to compare the mean scores of knowledge and attitude between the two communities. Bivariate and multivariate logistic regression models were employed to identify factors influencing caregivers’ knowledge and attitudes. Odds ratios (ORs) are presented with their 95% confidence intervals (CIs), and all analyses were performed at the 5% significance level (*p* < 0.05).

### Operational definitions

Knowledge: Caregivers’ understanding about diarrhea definition, cause, common clinical sign and symptoms, disease outcomes, management, and prevention towards their under-five children.

Attitude: Caregivers’ perception towards diarrhea among their under-five children.

Practice: Caregivers’ action towards the management of diarrhea towards their under-five children.

Good knowledge: Those caregivers who answered more than 75% of the knowledge questions correctly were considered to have good knowledge.

Average knowledge: Those mothers/caregivers who answered between half (50%) to three-fourth (75%) of the knowledge questions were considered as good knowledge.

Poor knowledge: Those caregivers who answered less than 50% of the knowledge questions correctly were considered to have poor knowledge.

Adequate knowledge: Those caregivers who answered 50% or more of the knowledge questions correctly were considered to have adequate knowledge.

Favorable attitudes: Those caregivers whose mean scores were above or equal to 50% of the attitude questions.

Unfavorable attitudes: Those mothers who scored less than the mean scores were below 50% of the attitude questions.

## Results

A total 1667 caregivers were interviewed and the response rate was 94%. The median age of respondents was 28.4 years (range 15–60 years). Half (834) of the caregivers had not received any formal education, 509 (30.5%) had attended primary school and 324 (19.4%) completed at least secondary education. In this study, 596 (35.8%) of under-five children had been ill with diarrhea during the 2 weeks prior to the survey and only 196 (32.9%) had been seen in health institutions. Seven hundred (42.0%) of the participants obtained health information about diarrhea in health care institutions, 563 (33.8%) from community health workers in their homes, and 60 (3.6%) through mass media. Nearly one fifth, 288 (17.3%), of the respondents never obtained health information from any external sources. Overall, 467 (28.0%) of the participants had received some health education from health workers within the last 3 months prior to the survey. The topics attended were HIV and STI by 126 (27.0%) caregivers, vaccination by 115 (24.6%), child nutrition by 92 (19.7%), diarrhea by 78 (16.7%), and water, sanitation and hygiene (WASH) by 43 (9.1%) caregivers, and 17 (3.6%) of caregivers attended other topics (Table 1).

**Table 1:** Characteristics of the caregivers among refugee and host communities in Gambella Region, Ethiopia, 2016

Variable		Refugee community		Host community	
		*n* = 854		*n* = 813	
		Freq.	%	Freq.	%
Caregiver sex					
Male		46	5.4	23	2.8
Female		808	94.6	790	97.2
Caregiver’s age group (year)					
≤ 24		232	27.2	263	32.4
25–34		447	52.3	436	53.6
≥ 35		175	20.5	114	14.0
Family size					
< 5		234	27.4	213	26.2
≥ 5		620	72.6	600	73.8
Caregiver’s marital status					
Married		735	86.1	709	87.2
Single		27	3.2	20	2.5
Divorced		40	4.7	37	4.6
Widowed		52	6.1	47	5.8
Ethnicity					
Agnuak		213	24.9	689	84.8
Nuer		641	75.1	90	11.1
Others		–	–	34	4.2
Caregiver education level					
No formal education		461	54.0	373	45.9
Primary school (grades 1 to 8)		264	30.9	245	30.1
Secondary school (grades 9 to 12)		63	7.4	113	13.9
Diploma and above		66	7.7	82	10.1
Described diarrhea signs and symptoms					
One sign or symptom		310	36.3	259	31.9
Two signs or symptoms		348	40.8	340	41.8
Three or more signs or symptoms		140	16.4	155	19.1
Did not describe		56	6.7	59	7.3
Usual source of health information					
Health institutions		358	41.9	342	42.1
Community health workers		286	33.5	277	34.1
Schools		35	4.1	21	2.6
Mass media (radio, TV)		30	3.5	30	3.7
Do not know		145	17.0	143	17.6
Health education attended within the last 3 months					
Yes		233	27.3	234	28.8
No		378	44.3	352	43.3
Do not remember		243	28.4	227	27.9
Health education topic attended last					
HIV and other STDs		56	23.7	70	29.8
Vaccination		52	22.0	63	26.8
Child feeding		50	21.2	42	17.9
Diarrhea		46	19.5	32	13.6
WASH		26	11.0	17	7.2
Others		6	2.5	11	4.7
Outcome of diarrhea described					
Not described		4	0.5	4	0.5
One outcome		116	13.6	92	11.2
Two or more outcome		734	85.9	717	88.2
Which treatments of diarrhea disease you know?					
Homemade fluids	Yes	83	9.7	44	5.4
	No	771	90.3	769	94.6
Oral rehydrating solution (ORS)	Yes	688	80.1	684	84.1
	No	166	19.9	129	15.9
Anti-diarrheal therapeutic medication such as antibiotics, Zn	Yes	401	47.0	331	40.7
	No	453	53.0	482	59.3
Herbal medicine	Yes	39	4.6	36	4.4
	No	815	95.4	777	95.6
Do not know	Yes	203	23.8	217	26.7
	No	651	76.2	596	73.3
ORS preparation procedure described					
Correct		268	31.4	195	24.0
Incorrect		586	68.6	618	76.0
Diarrhea prevention methods described					
One		47	5.5	33	4.1
Two or more		803	94.0	775	95.3
None		4	0.5	5	0.6

The overall mean knowledge score was 12.8 ± 3.1 (range 4–21). More than one third (633, 38.0%) of child caregivers had a low level of knowledge about diarrhea in under-five children. Out of 1034 (62.0%) participants with adequate knowledge, 997 (59.8%) had average knowledge while only 37 (2.2%) had good knowledge of childhood diarrhea. Four hundred fifty-four (27.2%) of the participants could properly define diarrhea. Nearly 34% (569) of the caregivers correctly identified one sign or symptom of diarrhea, followed by 688 (41.3%) who described two and 295 (17.7%) who listed three or more signs and symptoms. But 115 (6.9%) of the participants had difficulties in recognizing signs and symptoms of diarrhea. More than 99% (1656) of the caregivers explained at least one cause of diarrhea and 1333 (80.0%) of them could describe three or more causes of diarrhea.

Nearly all (95.5%) of child caregivers described a minimum of one undesirable outcome of diarrhea and 1452 (87.1%) of them listed two or more of its consequences. One thousand three hundred seventy-two (82.3%) of the respondents were familiar with ORS, 732 (43.9%) of the caregivers knew that diarrhea can be treated with antibiotics, 127 (7.6%) of them responded that it can be treated with homemade fluids, and 75 (4.5%), with traditional medicine. However, only 463 (27.8%) of the participants knew the correct ORS preparation procedure (one ORS sachet to 1 L of water) and 574 (34.4%) of them knew that prepared ORS should be discarded after 24 h. Moreover, 1578 (94.7%) of the respondents knew two or more ways of diarrhea prevention in under-five children.

The overall mean attitude score of the caregivers was 28.2 ± 5.1 (range 14–37). The majority (1274, 76.4%) of the respondents had a favorable attitude, and 393 (23.6%) had an unfavorable attitude on childhood diarrhea. About 22% (374) believed that diarrhea is normal in children. The middle proportion (916,54.9%) of the study participants either strongly agreed or agreed that exclusive breastfeeding is important in preventing childhood diarrhea (Table 2).

**Table 2:** Caregivers’ attitudes about diarrhea in under-five children in refugee and host communities in Gambella Region, Ethiopia, 2016

Variable	*N* = 1667 (%)				
	Strongly disagree	Disagree	Undecided	Agree	Strongly agree
Do you think that it is normal for children to get diarrhea regularly?	311 (18.7)	630 (37.8)	352 (21.1)	328 (19.7)	46 (2.8)
Do you think that hand washing with water and soap prevents diarrhea?	19 (1.1)	163 (9.8)	564 (33.8)	732 (43.9)	189 (11.3)
Do you think that hand washing before eating or feeding your child prevents diarrhea?	35 (2.1)	103 (6.2)	662 (39.7)	726 (43.6)	141 (8.5)
Do you think that hand washing after toilet or cleaning the child’s bottom prevent diarrhea?	13 (0.8)	269 (16.1)	454 (27.2)	804 (48.2)	127 (7.6)
Do you think that drinking clean water is important for the prevention of diarrhea?	11 (0.7)	257 (15.4)	389 (23.3)	725 (43.5)	285 (17.1)
Do you think that diarrhea can be treated with ORS?	16 (1.0)	157 (9.4)	606 (36.4)	787 (47.2)	101 (6.1)
Do you think that exclusive breastfeeding prevents diarrhea in children less than 6 months old?	17 (1.0)	102 (6.1)	632 (37.9)	717 (43.0)	199 (11.9)
Do you think that vaccination is harmful for children?	235 (14.1)	745 (44.7)	333 (20.0)	301 (18.1)	53 (3.2)

Variables with *p* value ≤ 0.25 in bivariate analysis were carried on and few of them appeared to be the independent predictors in the multivariate logistic model. Factors such as gender, age, and marital status were not associated with participants’ knowledge and attitudes. Knowledge was statistically higher among caregivers who had formal education (AOR, 1.3; 95% CI, 1.03–1.5) and obtained health information from a health care institution (AOR, 1.8; 95% CI, 1.28–2.3) compared to those who never accessed formal education and health education, respectively. Caregivers’ knowledge had an association with their attitude about diarrhea in under-five children (AOR, 2.5; 95% CI 1.6–3.8) (Table 3). Pearson’s correlation coefficient revealed that there was a positive correlation (*r =* 0.2, *p <* 0. 001) between knowledge and attitude scores. The independent *t* test also showed that there were no statistically significant differences in mean knowledge (*t* = 0.18) and attitude (*t* = 0.88) scores between the two communities.

**Table 3:** Factors associated with caregivers’ knowledge and attitudes on childhood diarrhea in refugee and host communities in Gambella Region, Ethiopia, 2016

Variables	Knowledge					Attitude				
	Poor	Adequate	COR (95% CI)	AOR (95% CI)	*p* value	Unfavorable	Favorable	COR (95% CI)	AOR (95% CI)	*p* value
Sex										
Male	32 (46.4)	37 (53.6)	1	1		17 (24.6)	52 (75.4)	1		
Female	601 (37.6)	997 (62.4)	1.4 (0.9–2.3)	1.2 (0.5–3.5)	0.67	376 (23.5)	1222 (76.5)	1.1 (0.6–1.9)	1.4 (0.4–4.2)	0.59
Age										
≤ 24	192 (38.8)	303 (61.2)	1	1		118 (23.8)	377 (76.2)	1	1	
25–34	343 (38.8)	570 (61.2)	1.0 (0.8–1.3)	0.86 (0.5–1.4)	0.51	207 (23.4)	676 (76.6)	1.0 (0.8–1.3)	0.9 (0.5–1.6)	0.8
≥ 35	98 (33.9)	191 (66.1)	1.2 (0.9–1.7)	1.8 (0.9–3.6)	0.08	68 (23.5)	221 (76.5)	0.98 (0.7–1.4)	0.7 (0.3–1.3)	0.27
Household size										
< 5	174 (38.9)	273 (61.1)	1	1		103 (23.1)	344 (76.9)	1	1	
≥ 5	459 (37.6)	761 (62.4)	1.1 (0.8–1.3)	1.5 (0.9–2.3)	0.082	290 (23.8)	930 (76.2)	0.96 (0.7–1.2)	0.9 (0.5–1.5)	0.72
Marital status										
Divorced	34 (44.2)	43 (55.2)	1	1		20 (26.0)	57 (74.0)	1	1	
Single	16 (34.0)	31 (66.0)		1.5 (0.7–3.3)	0.35	16 (34.0)	31 (66.0)	0.7 (0.3–1.5)	0.9 (0.2–4.4)	0.93
Married	543 (37.6)	901 (62.4)		1.3 (0.8–2.1)	0.29	332 (23.0)	1112 (77.0)	1.2 (0.7–2.0)	1.8 (0.7–1.6)	0.39
Widowed	40 (40.4)	59(59.6)		1.2 (0.6–2.1)	0.85	25 (25.3)	74 (24.7)	1.0 (0.5–2.1)	1.1 (0.3–3.8)	0.85
Educational level										
No formal education	339 (40.6)	495 (59.4)	1	1		198 (23.7)	636 (76.3)	1		
Formal education	294 (35.3)	539 (64.7)	1.3 (1.0–1.5)	1.3 (1.03–1.5)	0.022*	195 (23.2)	638 (76.6)	1.(0.8–1.3)	1.2 (0.8–1.8)	0.42
Usual sources of health information										
Health institutions	231 (33.0)	469 (67.0)	1.8 (1.3–2.3)	1.8 (1.3–2.3)	0.000*	152 (21.7)	548 (78.3)	1.4 (1.0–2.0)	1.2 (0.7–2.2)	0.53
Community health workers	225 (40.0)	338 (60.0)	1.3 (0.98–1.7)	1.3 (0.98–1.7)	0.068	131 (23.3)	432 (76.7)	1.3 (0.9–1.8)	1.4 (0.8–2.6)	0.25
Schools	21 (37.5)	35 (62.5)	1.4 (0.8–2.6)	1.5 (0.8–2.7)	0.185	13 (23.2)	43 (76.8)	1.3 (0.8–2.6)	–	–
Mass media (radio and television)	22 (36.7)	38 (63.3)	1.5 (0.8–2.7)	1.5 (0.8–2.7)	0.167	15 (25.0)	45 (75.0)	1.2 (0.6–2.3)	4.2 (0.9–19.9)	0.069
No source accessed	134 (46.5)	154 (53.5)	1	1		82 (28.5)	206 (71.5)	1	1	
Did you attend health education during the last 3 months										
Yes	167 (35.8)	300 (64.2)	1.1 (0.9–1.4)	1.3 (0.3–6.0)	0.76	112 (24.0)	355 (76.0)	1.0 (0.8–1.2)	1.2 (0.2–6.5)	0.86
No or not remember	466 (38.8)	734 (61.2)	1			281 (23.4)	919 (76.6)	1		
Health education topic attended last										
HIV and other STDs	52 (41.3)	74 (58.7)	1			26 (20.6)	100 (79.4)	1.4 (0.8–2.7)	1.5 (0.8–2.9)	0.22
Vaccination	40 (34.8)	75 (65.2)	1.3 (0.8–2.2)	1.4 (0.8–2.4)	0.21	29 (25.2)	86 (74.8)	1.1 (0.6–2.1)	1.1 (0.6–2.0)	0.82
Child feeding	35 (35.0)	57 (62.0)	1.1 (0.7–2.0)	1.2 (0.7–2.1)	0.53	25 (27.2)	67 (72.8)	1	1	
Diarrhea	26 (33.3)	52 (66.7)	1.4 (0.8–2.5)	1.5 (0.8–2.8)	0.16	20 (25.6)	58 (74.4)	1.1 (0.5–2.1)	1.3 (0.5–2.1)	0.92
WASH	13 (30.2)	30 (69.8)	1.6 (0.8–3.4)	1.8 (0.8–3.9)	0.13	10 (23.3)	33 (76.3)	1.2 (0.5–2.9)	1.2 (0.5–2.7)	0.75
Others	3 (17.7)	14 (82.3)	3.3 (0.9–12.0)	3.3 (0.9–12.5)	0.074	4 (23.5)	13 (76.5)	1.2 (0.4–4.1)	0.99 (0.3–3.4)	0.99
Caregivers’ knowledge										
Poor						193 (30.5)	440 (69.5)		1	
Adequate						200 (19.3)	834 (80.7)	1.8 (1.4–2.3)	2.5 (1.6–3.8)	0.00*

*The variable was significantly associated with caregivers’ knowledge or attitude about childhood diarrhea

## Discussion

Caregiver’s knowledge and attitude regarding causes, sign and symptoms, management, prevention, and control are very essential in reducing child morbidity and mortality due to diarrhea. So, assessing caregiver’s knowledge and attitude would be helpful in designing an effective health education strategy towards empowering them. This study revealed that more than one third (38%) of the caregivers had poor knowledge about childhood diarrhea, in both the refugee and host communities. This finding is in line with similar studies [30–32]. Caregivers with formal education (*p = 0.022*) had 30% better knowledge than those with no formal education. These findings are incongruent with a study done in Bangladesh [33] and in agreement with others [30]. This may be due to the fact that education augments parents’ knowledge of diarrhea [13, 34]. Only 27.2% of the caregivers could define diarrhea, less than those in studies in India, Iran, and Bangladesh [35–37]. This discrepancy may be due to different socioeconomic factors among study the participants. Nevertheless, the overall findings are similar to those of other studies [15, 38]. This could be due to the fact that half (50%) of the studied caregivers had no formal education and thus might have limited knowledge about diarrhea.

About 66% of them explained two or more symptoms of diarrhea. Majority (99.4%) of the caregivers could describe at least one cause of diarrhea that was similar with a study done in India [39]. The most commonly mentioned causes were eating unhygienic food 703 (42.2%) followed by eating with contaminated hands 621 (37.3%) and germs 141 (8.5%). Out of 596 diarrheic children, only 196 (32.9%) were taken by their caregivers to health facilities. Parents with poor knowledge are unlikely to go to hospitals because there is a positive association between knowledge and care-seeking behavior [40]. The percentage of caregivers who knew home-based fluids for treating diarrhea was very low in similar studies from other developing countries [41].

Although most of the participants were aware of ORS, our study reveals that there was poor knowledge of ORS preparation (26.4%) and its use (33.2%). This inferior knowledge of ORS preparation is in line with a study done in Nepal [14, 36] and contrasts with other studies [42, 43]. Nearly all (1578, 94.7%) of the caregivers knew two or more methods of prevention of diarrhea, more than those reported by other studies [37]. Caregivers who often obtained health information from health care institutions (*p < 0.001*) were 1.8 times more likely to have adequate knowledge than those who heard nothing about childhood diarrhea. This is due to the fact that health education improves human behavior and life style [44].

Our results also showed that a majority of the study participants had a favorable attitude against diarrhea, which is in agreement with other similar studies [45]. A considerable proportion (22.4%) of the caregivers perceived diarrhea to be a normal phenomenon occurring in growing children, which is in agreement with studies done in similar rural settings [21]. These wrong beliefs may reflect community culture in diarrhea prevention [46–49]. Nearly 17.7% of child caregivers considered that hand washing after using the toilet or cleaning a child’s bottom was not relevant to preventing diarrhea. Only one fourth of the caregivers believed that vaccination may be harmful to their child, as also reported by a study in India [50]. This may be due to lack of knowledge [51]. We found that caregivers’ attitude is significantly associated with their level of knowledge (*p* < 0.001) which is in turn affected by education, as caregivers who were highly literate were better informed about preventive practices. These findings are in agreement with the views by Rasania et al. and Bachrach and Gardner [43].

## Conclusion and recommendations

The mean knowledge and attitude scores among the two communities were analogous. The findings of this study indicate that significant numbers of caregivers had inadequate knowledge while some had unfavorable attitudes about diarrhea occurring in under-five children. Health promotion programs focusing on enhancing maternal knowledge and attitudes might have a protective effect on diarrhea and facilitate its management. Thus, designing and implementing an inclusive health education intervention focusing on uneducated child caregivers may be beneficial in improving their knowledge and attitudes towards lessening acute childhood diarrhea in such communities.

## Abbreviations

ARRA: Administration for Refugees and Returnees Affairs
UNHCR: United Nations High Commissioner for Refugees
WASH: Water, sanitation and hygiene
